# Impact of female students’ perceptions on behavioral intention to use video conferencing tools in COVID-19: Data of Vietnam

**DOI:** 10.1016/j.dib.2020.106142

**Published:** 2020-08-05

**Authors:** The-Hop Bui, Dinh-Hai Luong, Xuan-An Nguyen, Hong-Lien Nguyen, Thanh-Thuy Ngo

**Affiliations:** a Hanoi National University of Education, Vietnam; b Vietnam National Institute of Educational Sciences, Vietnam

**Keywords:** Video conferencing tools, Female students, Vietnam, Distance learning, COVID-19, Behavioral intention

## Abstract

This paper presents the dataset of a survey on Vietnamese female students’ behavioral intention to use video conferencing tools (VCTs; e.g. Zoom, Microsoft Teams or Google Hangout) in COVID-19 pandemic. The questionnaire was designed based on the Technology Acceptance Model according to the result of research conducted by [1] and [2]. The survey was conducted on an online platform using the questionnaire of 21 items to collect the information of respondents’ characteristics and their perception on computer self-efficacy, computer playfulness, COVID-19 context, behavioral intention to Use VCTs. The dataset contains 254 valid responses from female students who have been in distance learning in Vietnam. It can serve as a reference source for future studies on educational equity and for policy making on distance education.

**Specifications table**

**Table:** 

Subject	Social Sciences Education
Specific subject area	Online learning, Distance learning, Computer Self-efficacy, Computer playfulness, Equality, Higher Education
Type of data	Table
How data were acquired	Questionnaire
Data format	Raw Analyzed
Parameters for data collection	Participants who are female students of a university in Vietnam and are studying online through VCTs, decided to participate in the survey voluntarily
Description of data collection	Data were collected by snowball sampling and based on Internet platforms. The questionnaire was designed by Google Form and the survey was distributed for 10 days from April 14 to April 23. The data set includes 254 valid responses.
Data source location	Region: Asia Country: Vietnam Latitude and longitude: 21.028511, 105.804817
Data accessibility	Repository: Mendeley Data Data identification number: Direct URL to data: http://dx.doi.org/10.17632/6pcvfr6jg8

Value of the Data

• The dataset covered information of female students’ perceptions on using VCTs for online learning in Vietnam during the COVID-19 pandemic, and showed up the factors affecting their selection of VCTs in the future.
• Useful dataset for further comparing researches on distance learning issues among countries with diverse contexts or different stages of the COVID-19 pandemic.
• The dataset can be serve as a reference source for policy makers in making strategies for distance learning.
• The dataset is a reference source for studies on educational equity as well as gender equity in education.

## 1 Data description

The traditional way of teaching and learning in Vietnam, mainly based on face to face interactions, was interrupted when two positive cases of n-Covi were reported on January 23, 2020 [3], especially Vietnam began nationwide social distancing on April 1 [4]. On March 23, the Ministry of Education and Training issued the Document No. 988/BGDĐT-GDĐH [5] to guide higher education institutions to implement distance learning activities during the COVID-19 pandemic in order to ensure educational effectiveness and quality. Video conferencing tools (e.g. Zoom, Microsoft Teams or Google Hangout) as tools support distance learning are widely used in distance learning [6]. However, the process of converting the learning process from the traditional mode to the online method should be carefully prepared by higher education institutions to ensure the quality of training in accordance with the regulations of the Ministry of Education and Training [7]. In particular, ICT skills are an important factor affecting students’ learning [8], especially for female students [[9], [10]]. Designed based on the Technology Acceptance Model according to the result of research conducted by [1] and [2], computer self-efficacy, computer playfulness and social influence were external variables that have indirect effects on behavioral intention variables. This dataset focused on behavioral intention of Vietnamese female students in using VCTs, and related factors involving computer self-efficacy, computer playfulness and social influence variables.

The questionnaire includes 2 parts: the first section contains items collecting information about the respondent's characteristics, including university year, living area, VCTs used/using to study online, and devices using VCTs (see Table 1); The second part consists of items related to perceptions of respondents about Computer Self-efficacy, Computer playfulness, Context, and Behavioral Intention to Use (see Table 2). Table 3 shows more detailed results among variables. To complete the form, students spent about 15 min answering all questions. The results collected 254 valuable responses. Data raw and the questionnaire can be found in the Mendeley data repository [11].

**Table 1:** Respondents’ characteristics.

Characteristics	N	%
*University year*	*254*	*100.00*
1	72	28.35
2	117	46.06
3	41	16.14
4	22	8.66
5	2	0.79
*Area*	*254*	*100.00*
Rural	120	47.24
Urban	133	52.36
*Number of VCT*	*254*	*100.00*
1	129	50.79
2	116	45.67
3	7	2.76
4	2	0.79
*VCT*	*254*	*100.00*
Microsoft Teams	14	5.51
Google Meet	211	83.07
Zoom	144	56.69
Others	34	13.39
*Number of devices*	*254*	*100.00*
1	109	42.91
2	135	53.15
3	9	3.54
4	1	0.39
*Type of devices*	*254*	*100.00*
Laptop	179	70.47
Smartphone	204	80.31
Desktop	22	8.66
Tablet	7	2.76

**Table 2:** Descriptive results of participants’ responses.

Variables		N	Min	Max	Mean	Std. Deviation
*Computer Self-efficacy (CSE) (Cronbach's Alpha = 0.92)*						

CSE1	I feel confident in the utilization of VCT even when no one is there for assistance.	254	1	5	3.44	1.03
CSE2	I have sufficient skills to use VCT.	254	1	5	3.65	0.95
CSE3	I feel confident when using the VCT even if I have only the online instructions.	254	1	5	3.54	0.98
CSE4	I feel confident when using VCT features.	254	1	5	3.55	0.92
CSE5	I feel confident when using the online learning content in VCT.	254	1	5	3.50	0.92

*Computer playfulness (CP) (Cronbach's Alpha = 0.88)*						

CP1	I feel that VCT is enjoyable no matter what the usage purposes are.	254	1	5	3.15	0.864
CP2	I feel that VCT helps me to improve my creativity.	254	1	5	2.98	0.913
CP3	I feel that VCT helps me to improve my imagination by obtaining information.	254	1	5	2.95	0.905
CP4	I feel that I can have a variety of experiences without any interference.	254	1	5	3.05	0.91

*Context (CON) (Cronbach's Alpha = 0.78)*						

CON1	I feel that VCT is a temporary solution during the Covid-19 pandemic	254	1	5	3.85	.946
CON2	I feel that VCT is a mandatory solution for students to continue the learning process during the Covid-19 pandemic	254	1	5	3.94	.980
CON3	I feel that VCT made the learning space narrowing	254	1	5	3.88	.979

*Behavioral Intention to Use (BI) (Cronbach's Alpha = 0.89)*						

BI1	I will make use of VCT regularly in the forthcoming time.	254	1	5	3.26	0.938
BI2	I intend to make use of functions of VCT for providing assistance to my academic activities.	254	1	5	3.36	0.882
BI3	I will give out my recommendation to others to use VCT.	254	1	5	3.23	0.892
BI4	I will use VCT on a regular basis in the future.	254	1	5	3.10	0.904

**Table 3:** Correlations between variables and behavioral intention to use variable.

Variable		Behavioral intention to use			
		*BI1*	*BI2*	*BI3*	*BI4*
*Respondent characteristics*					

DEM1	University year	0.027	-0.049	0.094	−0.026
DEM2	Area	0.065	0.032	0.056	−0.03
DEM3	Number of VCT	−0.006	0.014	−0.078	0.064
DEM4	Number of devices	0.018	−0.03	−0.033	0.076

*Computer Self-efficacy*					

CSE1	I feel confident in the utilization of VCT even when no one is there for assistance.	0.351⁎⁎	0.339⁎⁎	0.347⁎⁎	0.276⁎⁎
CSE2	I have sufficient skills to use VCT.	0.292⁎⁎	0.280⁎⁎	0.346⁎⁎	0.234⁎⁎
CSE3	I feel confident when using the VCT even if I have only the online instructions.	0.273⁎⁎	0.225⁎⁎	0.302⁎⁎	0.211⁎⁎
CSE4	I feel confident when using VCT features.	0.369⁎⁎	0.311⁎⁎	0.347⁎⁎	0.308⁎⁎
CSE5	I feel confident when using the online learning content in VCT.	0.514⁎⁎	0.459⁎⁎	0.481⁎⁎	0.412⁎⁎

*Computer playfulness*					

CP1	I feel that VCT is enjoyable no matter what the usage purposes are.	0.603⁎⁎	0.523⁎⁎	0.520⁎⁎	0.548⁎⁎
CP2	I feel that VCT helps me to improve my creativity.	0.450⁎⁎	0.492⁎⁎	0.437⁎⁎	0.507⁎⁎
CP3	I feel that VCT helps me to improve my imagination by obtaining information.	0.478⁎⁎	0.446⁎⁎	0.497⁎⁎	0.509⁎⁎
CP4	I feel that I can have a variety of experiences without any interference.	0.480⁎⁎	0.446⁎⁎	0.434⁎⁎	0.557⁎⁎

*Context*					

CON1	I feel that VCT is a temporary solution during the Covid-19 pandemic	0.453⁎⁎	0.390⁎⁎	0.334⁎⁎	0.333⁎⁎
CON2	I feel that VCT is a mandatory solution for students to continue the learning process during the Covid-19 pandemic	0.197⁎⁎	0.216⁎⁎	0.198⁎⁎	0.152*
CON3	I feel that VCT made the learning space narrowing	0.346⁎⁎	0.325⁎⁎	0.247⁎⁎	0.254⁎⁎

⁎⁎ Correlation is significant at the 0.01 level (2-tailed).

The dataset includes five groups of variables: (1) Respondents’ characteristics (see Table 1); (2) Female students’ perceptions of their computer self-efficacy; (3) Female students’ perceptions of their Computer playfulness; (4) Female students’ perceptions of their Behavioral Intention to Use VCTs; and (5) Female students’ perceptions of using VCTs for distance learning in this context (see Table 2).

## 2 Experimental design, materials and methods

The survey have been conducted via internet for 10 days from April 14 to April 23 when Vietnam was adopting social distancing and promoting distance learning whole the country. The survey form was designed by the Google Forms application, including 21 items, of which 4 items were about the characteristics of the survey participants, 17 left items, which were designed on a 5-point Likert scale (1: totally disagree; 2: somewhat disagree; 3: Neither agree or disagree; 4: somewhat agree; 5: totally agree), focused on 4 factors: Computer self-efficacy, Computer playfulness, Context, and Behavioral intention to Use. Online survey was the only available option at this time and the snowball sampling method was applied. Participants were female students who decided whether or not to participate in the survey as well as introduce their friends by themselves. The form was distributed to the email address of female students which were provided by their lecturers. All items in the survey were required to be completed, and the data collected do not contain missing data. All of respondents' responses stored in Google Forms were exported as Master Excels (csv file) to import to SPSS 20. Before performing the analysis methods, the variables were renamed (see Table 2) and the data were checked for the validity of each case, no cases were excluded by the respondents provided constant value for all items. The final dataset had 254 valid cases.

Based on this dataset, further researches are able to conduct studies on the relationship between perceptions of learners on Behavioral intention to use technology towards distance learning in universities [12], open learning policy making [13] or blended learning [14].

## Ethics statement

The authors kept to all ethical concerns during the data gathering process. The authors ensured that all respondents' information is used for research purposes and is absolutely confidential.

## Declaration of Competing Interest

The authors declare that they have no known competing financial interests or personal relationships which have, or could be perceived to have, influenced the work reported in this article.
